# XP-J, a ninth xeroderma pigmentosum complementation group, results from mutations in *GTF2H4*, encoding TFIIH-p52 subunit

**DOI:** 10.1172/JCI195731

**Published:** 2025-09-09

**Authors:** Hiva Fassihi, Shehla Mohammed, Yuka Nakazawa, Heather Fawcett, Sally Turner, Joanne Palfrey, Isabel Garrood, Adesoji Abiona, Ana M.S. Morley, Mayuko Shimada, Kana Kato, Alan R. Lehmann, Tomoo Ogi

**Affiliations:** 1 National Xeroderma Pigmentosum Service, Rare Disease Centre, Guy’s and St Thomas’ NHS Trust, London, United Kingdom.; 2 Department of Molecular Genetics, Nagoya University Graduate School of Medicine, Nagoya, Japan.; 3 Department of Genetics, Research Institute of Environmental Medicine, Nagoya University, Nagoya, Japan.; 4 Genome Damage and Stability Centre, University of Sussex, Brighton, United Kingdom.; 5 Rare Disease Genome Centre, Nagoya University, Nagoya, Japan.

**Keywords:** Dermatology, Genetics, DNA repair, Genetic diseases, Monogenic diseases

**To the editor:** Xeroderma pigmentosum (XP) is a genodermatosis, characterised by sun-induced pigmentation and increased skin-cancer risk due to defective DNA repair (1, 2). XP was first described by Kaposi and Hebra in 1874, but its cellular- and molecular-basis began to emerge in the 1960s. Cell-fusion complementation assays revealed genetic heterogeneity in XP by showing that fusion of XP-fibroblasts from different patients restored DNA repair (2). This led to the identification of complementation-groups XP-A to XP-G; groups H and I were later discovered to be due to errors (Supplemental Table 1; supplemental material available online with this article; https://doi.org/10.1172/JCI195731DS1) (1, 2). Late 20th-century molecular genetics identified the XP-causative genes, XPA-XPG/ERCC5 (Supplemental Table 1) (1, 2). The majority of patients with XP lack nucleotide excision repair (NER), which removes photolesions from the genome (1). NER-defective XP results from impaired photodamage recognition (DDB2/XPE, XPC) or defective incision involving DNA-repair/transcription-factor TFIIH, XPA, and endonucleases. XPB/p89 and XPD are ATPase/helicases of TFIIH, while XPF-ERCC1 and XPG act as 5’- and 3’-endonucleases, respectively (3, 4). XP-variant (XP-V), an NER-proficient form, described in 1971, was later linked to mutations in *POLH*, encoding DNA polymerase η that bypasses UV photolesions. To date, all XP cases have been classified into eight complementation groups (XP-A to G and V) (1, 2). Here, we report a patient assigned to a ninth complementation-group, XP-J, identified 50-years after the others. The patient exhibits NER-defective XP-features without signs of other NER disorders, such as Cockayne syndrome (CS) or trichothiodystrophy (TTD) (1). Pathogenic mutations in *GTF2H4*, encoding the TFIIH-p52 subunit (5), likely disrupt its interaction with p8 (4), whose deficiency causes severe TTD-A (1).

XP140BR, a 6-year-old Caucasian girl diagnosed with XP, shows severe photosensitivity, progressive exposed-site lentigines, microcephaly, and mild developmental delay (Figure 1; case report in Supplemental materials). At three months, she developed severe sunburn with minimal sun exposure, followed by recurrent burns despite sun protection. Lentigines appeared on exposed skin (Figure 1, A–D). Phototesting showed a markedly reduced minimum-erythema-dose (MED) (UVB < 0.03 J/cm^2^), causing persistent erythema and blistering (Figure 1E). She showed no photophobia or ocular changes and had motor and speech delays. Notably, she lacked any defining features of CS or TTD (1).

We first measured UV-induced DNA repair. Global genome NER (GG-NER) deficiency is typical of all NER-defective XP but not CS cases, while transcription-coupled NER (TC-NER) is impaired in CS, TTD, and most XP-groups except XP-C and XP-E (1, 2). Unscheduled DNA synthesis (UDS) measures GG-NER by quantifying repair DNA synthesis; TC-NER is evaluated by the recovery of RNA synthesis (RRS) after DNA damage (2). XP140BR showed defects in both, indicating GG- and TC-NER deficiencies (Figure 1, F and G), while transcription was not affected (Supporting data values). We presumed the patient carries mutations in one of the NER genes. Lentivirus UDS-complementation assays with each NER-cDNA failed to restore the UDS-defect, indicating that XP140BR does not belong to any known XP complementation group (Figure 1H).

We performed whole-genome-sequencing and identified potentially pathogenic variants in *GTF2H4*, encoding the TFIIH-p52 subunit (Figure 1I). The first variant, c.138-1G>A, is located at the intron 2–exon 3 boundary, leading to skipping of exon 3. This results in an in-frame deletion (p52Δ47–81aa); however, this product is unstable and undetectable by immunoblot. The second variant, c.1203_1204delinsGAG, is a frameshift-mutation located in exon 13, resulting in a p.Phe403Valfs*3 C-terminal truncation (p52ΔC). The p52ΔC protein is stably expressed and the entire TFIIH complex remains stable, although the amount is slightly reduced (Figure 1J). To determine whether these *GTF2H4* variants are XP causative, we performed a UDS lentivirus–complementation assay. The UDS-defect was restored when *GTF2H4*-cDNA was expressed (Figure 1H). Mutations in *GTF2H4* therefore cause NER-defective XP, and XP140BR defines a ninth XP complementation-group. We designate this as XP-J, since XP-H and XP-I were later found to be identical to XP-D and XP-C, respectively. This will prevent confusion with earlier classifications (Supplemental Table 1).

In summary, we designate an XP patient with mutations in *GTF2H4* as XP-J, establishing it as a ninth XP complementation group. This marks the first identification of a new XP complementation group in five decades, since XP-G (1973), and 25-years, since the cloning of *POLH* for XP-V (1999) (references in Supplemental Table 1). This is the first TFIIH-related disorder reported since TTD-A, caused by mutations in *GTF2H5* encoding p8 (2004). The patient with XP-J exhibited definitive NER-deficient XP features without neurological symptoms. Notably, the patient lacked TTD features, despite TFIIH-p52 mutations that are expected to disrupt p52 interaction with p8, deficiencies in which cause TTD-A.

In the accompanying paper (6), we describe how truncation of the p52 C-terminal domain, required for the p52-p8 interaction, can compensate for p8 loss and helps prevent the development of severe TTD-A. In that paper, we also present a therapeutic approach using antisense oligonucleotides (ASOs) for mitigating TTD-A by inducing p52 C-terminal truncation, aiming to stabilise the TFIIH complex.

## Supplementary Material

Supplemental data

Unedited blot and gel images

Supporting data values

## Figures and Tables

**Figure 1:**
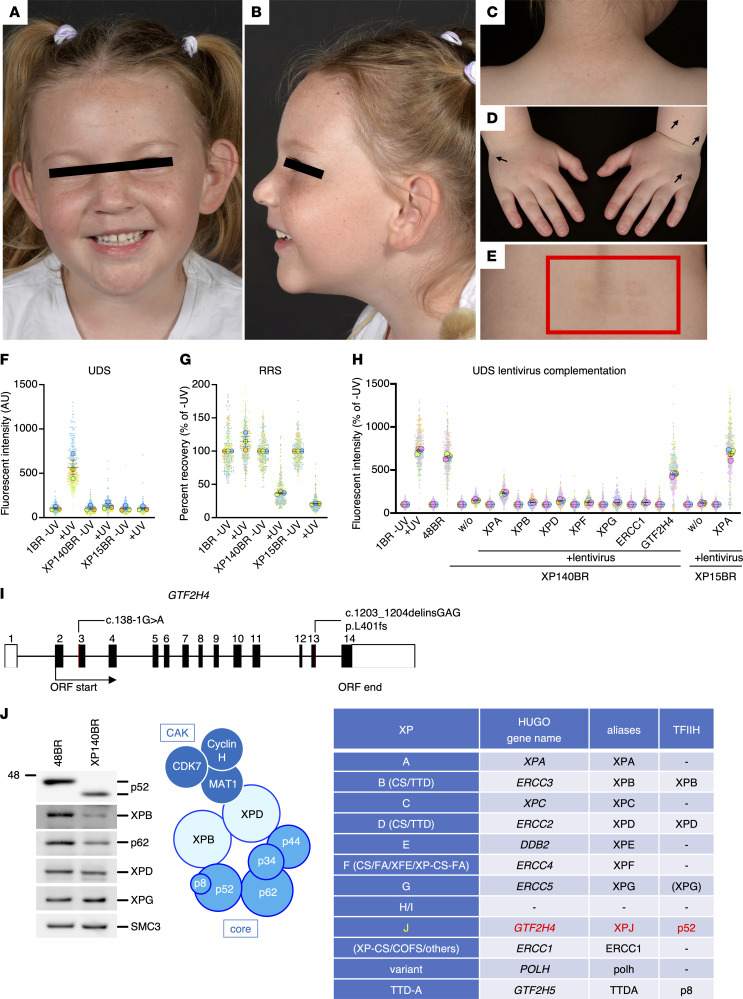
XP140BR: 6-year-old girl with XP-J. (**A**–**D**) Microcephaly without enophthalmos; lentigines on face, posterior neck, and dorsum of hands (black arrows). (**E**) Scar from blistering sunburn 6 months after UVB-phototesting (red box). (**F** and **G**) XP140BR cells are NER-deficient. 1BR (normal control), XP140BR (XP-J), XP15BR (XP-A); 20J/m^2^ UVC-irradiation (**F**). 15J/m^2^ UVC-irradiation (**G**). (**H**) XP140BR is not complemented by known XP cDNAs, but is rescued by *GTF2H4*. 20J/m^2^ UVC-irradiation. Bars and error bars represent means and SEM, respectively, of experiments (*n* = 3–4, as indicated by the colored circles and their corresponding plots). (**I**) *GTF2H4* structure and mutations. (**J**) TFIIH-p52 subunit, encoded by *GTF2H4*, is C-terminally truncated in the patient, but still forms a stable complex. Left, immunoblots of major TFIIH-subunits (SMC3, loading control); Middle, schematic of TFIIH-subunit interactions; Right, XP genes associated with TFIIH-subunits (references in Supplemental Table 1).

## Version 1 09/09/2025

In-Press Preview

## Version 2 11/17/2025

Electronic publication
